# Holistic processing of Chinese characters in college students with dyslexia

**DOI:** 10.1038/s41598-021-81553-5

**Published:** 2021-01-21

**Authors:** Ricky Van-yip Tso, Ronald Tsz-chung Chan, Yin-fei Chan, Dan Lin

**Affiliations:** 1 Department of Psychology, The Education University of Hong Kong, Hong Kong, China; 2 Psychological Assessment and Clinical Research Unit, The Education University of Hong Kong, Hong Kong, China

**Keywords:** Neurodevelopmental disorders, Disability, Psychology

## Abstract

Expert face recognition has long been marked by holistic processing. Hence, due to the many visual properties shared between face perception and Chinese characters, it has been suggested that Chinese character recognition may induce stronger holistic processing in expert readers than in novices. However, there have been different viewpoints presented about Chinese character recognition, one of which suggests that expertise in this skill involved reduced holistic processing which may be modulated by writing experiences/performances. In this study we examined holistic processing in Chinese character recognition in adults with and without dyslexia, using the complete composite paradigm. Our results showed that the adults with dyslexia recognized Chinese characters with a stronger holistic processing effect than the typical controls. It seems that those with dyslexia relied overly on the visual spatial information of characters and showed deficits in attending selectively to their components when processing Chinese characters, which hindered the development of expert reading and writing skills. This effect was in contrast to previous perceptual expertise studies in which reduced holistic processing marked deficits in face/visual object recognition. This study is also the first to show that Chinese adults with dyslexia had persistent below average performances in Chinese literacy.

## Introduction

**Figure 1:**
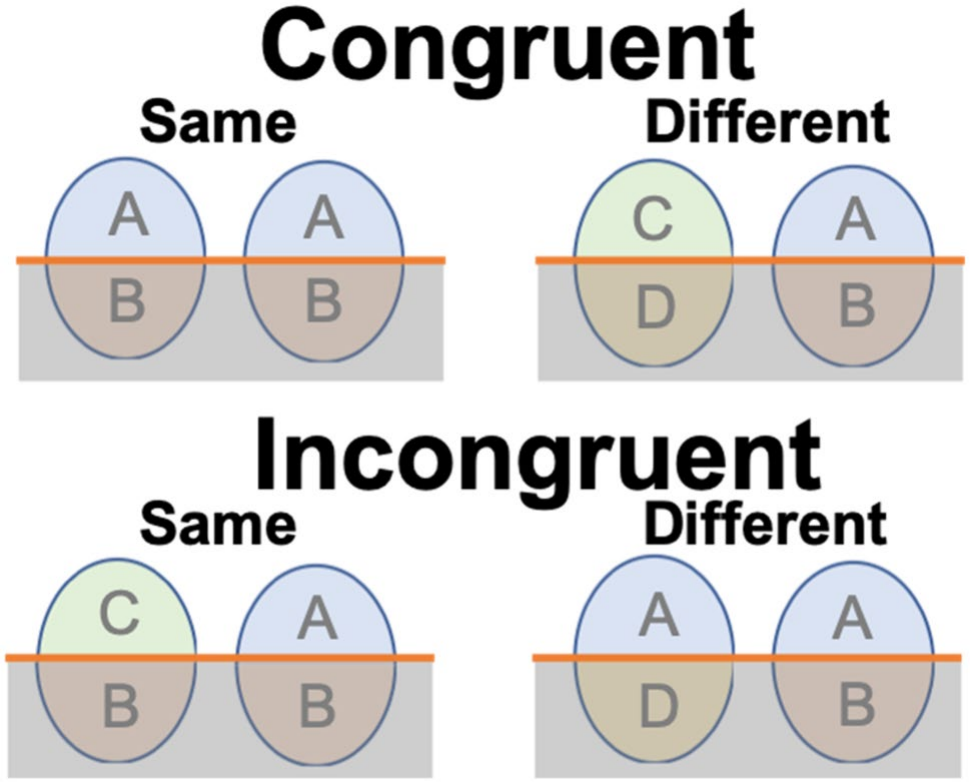
Complete composite paradigm to measure holistic processing. In each trial, participants were cued to attend to the top or bottom half of each stimulus pair and judged whether the attended halves were the same or different (attended halves shaded in grey in the figure). Holistic processing was demonstrated by a lower performance level in the incongruent condition than in the congruent condition, induced by the interference of the irrelevant halves. This paradigm has been widely adopted to measure for holistic processing in face recognition^10^.

Holistic processing, according to Gestalt psychology, is defined as the processing of the whole of sensory inputs that are qualitatively different from their separable parts^1,2^. Considered to be a perceptual marker of visual expertise, holistic processing—a phenomenon found commonly in face recognition—has been researched and documented widely^3^. Other than its use in face recognition, some studies have suggested that it is also used in other types of visual objects in which within-category individualization is required^4^. For example, an association has been found between holistic processing and expert recognition of visual objects after participants were trained to recognize novel synthetic stimuli^5^. It is also found that training to individualize an artificial object type increased holistic processing^6^. A paradigm which can demonstrate holistic processing is the composite paradigm, which demonstrates a type of visual perception in which both featural and configural information are integrated and processed as a Gestalt^7^ (see Fig. 1). The composite paradigm demonstrates holistic processing by inducing the composite face illusion: two identical top halves from a face-pair are more likely to be perceived as different when the bottom halves of the face-pair are from different faces^8^. The above illusion suggests a failure in selective attention to facial parts as a result of holistic processing: viewers obligatorily pay attention to all features of a face as a whole during face perception^9^.

A point to note is that another commonly used paradigm, the part-whole task, can also demonstrate holistic processing^7,11^: in the “whole-trials”, participants were first shown a face, after which 2 faces appear simultaneously on the screen that differed in only one facial feature (e.g. the nose), with one face being identical to the previous face stimuli; in the “part-trials”, two isolated facial feature of the same part (e.g. nose) appear, with only one stimuli identical to that appeared in the previously shown face. Participants judged which face or facial feature appeared in the previous facial stimuli. Participants generally identified the whole faces with a higher accuracy than when only facial parts were shown—holistic processing is measured by the discrepancy between the performance in the whole- and part-trials. DeGutis et al. (2013) has shown that the part-whole paradigms are correlated strongly with the composite paradigm, which both correlates with face recognition ability when regression was used to measure holistic processing by controlling the variance in the baseline conditions^12^. However, the part-whole paradigm is heavily influenced by memory^3^. Moreover, although paradigms that demonstrates inversion effect (i.e. upside-down stimuli impairs recognition performance) were suggested to demonstrate holistic processing, inversion effect demonstrates perceptual learning and novelty effect^13^ rather than the type of holistic processing that integrates both featural and configural information of visual stimuli^7^. Furthermore, the underlying mechanism of inversion effect is unclear and does not correlate with measures from the part-whole and composite paradigms^14^. Therefore, this study investigates the composite paradigm over the part-whole paradigm to reduce the demand of memory since this study aims at examining perceptual processes; while the composite effect was examined rather than inversion effect as it is more incline with our definition of holistic processing—the obligatory attention to all parts of a whole.

### Holistic processing in Chinese character recognition

Intuitively, Chinese characters may seem to belong to a distinct class of visual stimuli, different from that to which faces belong. For example, while the same radicals and strokes can appear in different positions in a character, the single features (such as eyes and mouth) of each individual face differ but appear in the same positions. While Chinese characters can take forms in more than 10 types of configurations (including top–bottom and left–right^15^, faces are always symmetrical and configurally top–bottom. However, there are similar visual properties shared between Chinese character and face recognition. Both are always viewed upright. Chinese characters are configurally squared and homogenous—with each character a grapheme represents a morpheme^15^. Moreover, Chinese characters are formed by more than 200 basic Chinese character stroke patterns, combined by strokes as the basic units^16^. Face configurations are also homogenous, with different individual faces formed by endless combinations of facial features. Different faces can be differentiated and recognized regardless of their expressions, and similarly over 3000 different Chinese characters can be recognized by a typical literate, irrespective of fonts^17^. Hence, Chinese characters should theoretically induce a similar (holistic) perceptual expertise effect as faces due to the similarities between individualization of different faces and naming individual Chinese characters^18^. Indeed, common perceptual markers of observed in faces perception such as the inversion has been demonstrated in expert readers in word recognition^19–21^.

However, whether holistic processing measured by the composite paradigm is an expertise marker in words recognition is inconclusive. For example, expertise in English word recognition is characterized by a stronger composite effect in native English readers when compared with English-as-a-second language readers^22^. Another study found that holistic processing is modulated by phonological regularities in Portuguese words (an alphabetic writing system)—phonologically consistent words elicited a composite effect, while phonologically inconsistent words did not^23,24^. Similar effects have been found in Chinese character recognition. For example, Wong et al. (2012) found composite effect in Chinese character recognition for both expert and novices^25^. However, while expert showed composite effect for real characters but not noncharacters, novices showed composite effect for both real and noncharacters, suggesting that holistic processing is a general hallmark for word recognition^25^. The finding that novices showed composite effect even for noncharacters suggested that the complex pattern of Chinese characters are less readily decomposed into parts as novel stimuli^25^. This is also reflected in Hsiao and Cottrell’s study that showed a stronger composite effect in Chinese character recognition in novices than in expert readers. Perhaps reduced holistic effect shown by the expert readers may be explained by writing performances^26^. Tso et al. (2014) found that expert readers with limited writing performances (Limited-writers) used more holistic processing than the novices, while the expert readers with typical writing abilities (Writers) showed a reduced holistic effect^26^. They found that while both Limited-writers and Writers had comparable character naming accuracy, the Writers had significantly faster response times, suggesting the Writers had a more efficient character processing approach than the Limited-writers^26^. Hence, although holistic processing is consistently shown to be a hallmark of expert word recognition, it seems that expertise in Chinese character writing involves the ability to attend to and recognize character components selectively, as demonstrated in reduced holistic effects. This finding is also consistent with the study in which artists with face-drawing experience demonstrated reduced holistic processing in face recognition when compared with people without such experience^27^. These findings altogether suggest that holistic processing demonstrates an analogous pattern in the development of visual expertise in both face and Chinese character recognition, supporting a modulating role of writing/drawing abilities in holistic processing.

### Holistic processing in the population with special needs

Developmental dyslexia (dyslexia) is a specific learning disorder (SpLD) characterized by persistent difficulties in reading and/or spelling. Much work has been done to understand the cognitive mechanisms underlying reading difficulties. However, reading is a complex cognitive process that involves various metalinguistic skills, particularly skills in phonological processing^28^. Moreover, in recent decades there has been growing academic interest in the basic visual cognitive skills of reading and its relationship with reading difficulties, and various reports have investigated the possible role of these skills in reading processes^29^. Attention to the spatial location of the visual-orthographic information within a written word is important for reading ability in typical readers learning to read words^30^. Indeed, other visual perceptual abnormalities in word recognition has been also been found in people with developmental dyslexia. For example, the inversion and word superiority effects that have been commonly observes in typical readers were absent or reduced in dyslexic readers^31^.

While the majority of research in the literature focuses on alphabetic scripts, there is a growing number of studies of visual-orthographic processing and its association with reading processes in Chinese reading development^32–35^. It has been suggested that visual processing plays a more vital role in the Chinese writing system, generally considered to be a logographic script, than in alphabetic reading, given the relatively complex visual features of Chinese characters^36^. Indeed, Chinese-word reading has a strong basis in visual-orthographic processing demonstrated by writing and copying abilities due to the deep-rooted practice of learning Chinese characters through copying, which shapes both the perceptual and neural representations for word recognition^37–42^.

Whereas English dyslexia is generally associated with core deficits in phonological skills, dyslexia in the Chinese language has been shown to be characterized by a visuospatial deficit^36,43,44^. Consistently, different brain abnormalities characterize developmental dyslexia in an alphabetic script and in the Chinese writing system^36,45^. While dyslexia in alphabetic languages is characterized by neurological deficits related to phonological skills (e.g. left temporoparietal regions), in Chinese it is associated more with abnormalities in regions that are responsible for orthography or visuospatial processing (e.g. middle frontal regions). Children with Chinese reading difficulties are often observed to have a marked discrepancy between reading and writing abilities, due to writing in Chinese being a more resource-intensive process than writing in alphabetic languages^46^. The above findings all suggest a strong visual-spatial basis in the process of Chinese character recognition and its strong correlations with writing abilities. Since holistic processing is a type of visual-spatial process modulated by writing/drawing (motor) experiences, this study examined the role of holistic processing and its relationship with reading abilities in Chinese college students diagnosed with developmental dyslexia^26,27^. The complete composite paradigm, identical to the design as shown in Fig. 1 but with Chinese characters in place of face stimuli, was adopted to measure holistic processing in Chinese character recognition^17,26^.

Moreover, perceptual abnormalities in holistic processing of visual stimuli have been shown to occur in populations with cognitive disabilities when compared with typical controls. For example, reduced holistic processing in face recognition has been associated with face-recognition difficulties in patients with prosopagnosia^47^. It also marks a cognitive deficit in people with autism, who are often found to have poorer abilities in face and facial expression recognition than the general population^48^. However, since reduced holistic processing has been shown to mark the expertise profiles of expert Chinese readers, perhaps analytic processing is required for attending to Chinese character components for expert-level recognition and writing^17^. Readers with developmental dyslexia often have a deficiency in orthographic sensitivity of the internal structures of Chinese characters; hence, contrary to a lack of holistic processing being the marker of cognitive deficits in face perception, people with dyslexia may instead process Chinese characters more holistically as the components and radicals in a Chinese character may look more visually inseparable to them^49^. Furthermore, research in western contexts has shown that adults with dyslexia demonstrated persistent difficulties in reading, as well as weaker literacy-related cognitive abilities than typically developing adults^50^. To date, we are unable to find studies that document developmental dyslexia in the Chinese language for the both the adult population and college students from the perspective of visual perception, this study is perhaps the first to add this documentation to the literature.

## Results

### Literacy abilities and non-verbal intelligence (dyslexics vs control)

Separate t-tests were carried out to examine the effect of group (Dyslexics vs Control) on each literacy test. In the 9-item Raven’s test, we found that the dyslexic and control groups did not differ significantly, *t*(42) = 1.001, *p* = 0.323, *d* = 0.31, which suggests that both groups had similar non-verbal intelligence. Nevertheless, participants in the control group had outperformed those with dyslexia in Chinese word-reading, *t*(42) = 2.973, *p* = 0.005, *d* = 0.92, one-minute word reading, *t*(1, 42) = 3.101, *p* = 0.003, *d* = 0.96, Chinese word dictation task, *t*(1, 42) = 4.262, *p* < 0.001, *d* = 1.32. and rapid digit naming, *t*(1, 42) = 4.531, *p* < 0.001, *d* = 1.40. These results suggested that our college students with dyslexia generally had poorer literacy abilities than their control counterparts. See Table 1 for a summary.

**Table 1:** Summary of the scores of Chinese literacy performance and non-verbal IQ (9-item Raven’s) in college students with and without dyslexia.

	Control Mean (SE)	Dyslexics Mean (SE)
Chinese word-reading	114.8 (.67)	107.5 (2.35)
Chinese one-minute word reading	101.66 (5.37)	80.52 (4.20)
Chinese dictation	75.27 (1.28)	57.41 (3.99)
Rapid digit naming	11.72 (.61)	16.20 (.77)
Non-verbal IQ (9-item Raven’s)	6.45 (.50)	5.73 (.52)

### Holistic processing

#### A′

We next examined the ability to process Chinese characters holistically, comparing participants with and without dyslexia. We first conducted a 2 (congruency: congruent vs. incongruent) × 2 (alignment: aligned vs misaligned) × 2 (group: dyslexics vs. control) repeated-measures ANOVA on *A′*, which showed a main effect of congruency, *F*(1, 42) = 19.956 *p* < 0.001, *η*_*p*_^2^ = 0.322, a main effect of group, *F*(1, 42) = 14.429, *p* < 0.001, *η*_*p*_^2^ = 0.256, and an interaction between congruency and group, *F*(1, 42) = 7.915, *p* = 0.007, *η*_*p*_^2^ = 0.159. However, there is no main effect of alignment, *F*(1, 42) = 0.879, *p* = 0.354, interaction between alignment and group, *F*(1, 42) = 2.292, *p* = 0.138, nor interaction between congruency, alignment and group, *F*(1, 42) = 0.824, *p* = 0.369. A significant interaction effect was found between alignment and congruency, *F*(1, 42) = 4.464, *p* = 0.0419, *η*_*p*_^2^ = 0.096.

A post-doc 2 (congruency: congruent vs. incongruent) × 2 (alignment: aligned vs misaligned) repeated-measures ANOVA was conducted on *A′* in participants with and without dyslexia separately. In participants with dyslexia, a significant main effect was found for congruency, *F*(1, 21) = 13.52, *p* = 0.001, *η*_*p*_^2^ = 0.392, but no main effect of alignment, *F*(1, 21) = 1.084, *p* = 0.1.94, nor an interaction between congruency and alignment was found, *F*(1, 21) = 2.606, *p* = 0.121. In typical readers, a significant main effect was found for both congruency, *F*(1, 21) = 6.431, *p* = 0.019, *η*_*p*_^2^ = 0.234, and alignment, *F*(1, 21) = 16.146, *p* = 0.001, *η*_*p*_^2^ = 0.435; however, no interaction between congruency and alignment was found, *F*(1, 21) = 2.430, *p* = 0.134. Post-doc *t*-tests showed that participants with dyslexia responded with a larger performance level in the in the aligned than in the misaligned conditions in incongruent trials, *t*(21) = 2.201, *p* = 0.039, *d* = 0.47, whereas *A′* in the aligned and misaligned conditions were similar in the congruent trials, *t*(21) = 0.426, *p* = 0.674. In contrasts, the typical readers had a responded with similar performance levels in aligned conditions and misaligned conditions in both congruent, t(21) = 0.026, p = 0.970 and misaligned conditions t(21) = 1.050, *p* = 0.305. Both dyslexic and typical readers showed a larger *A′* in congruent than incongruent trials in both aligned [*t*(21) = 4.182, *p* < 0.001, *d* = 0.88, and *t*(21) = 4.695, *p* < 0.001, *d* = 1.00, respectively] and misaligned conditions [*t*(21) = 2.749, *p* = 0.012, *d* = 0.59, and *t*(21) = 3.454, *p* = 0.002, *d* = 0.74, respectively].

##### Response time

We then conducted a 2 (congruency: congruent vs. incongruent) × 2 (alignment: aligned vs misaligned) × 2 (group: dyslexics vs. control) repeated-measures ANOVA on Response Time (RT) for correct responses, which showed a significant main effect of congruency, *F*(1, 42) = 17.492, *p* < 0.001, *η*_*p*_^2^ = 0.294, a main effect of alignment, *F*(1, 42) = 6.720, *p* = 0.013, *η*_*p*_^2^ = 0.138, an interaction between congruency and alignment, *F*(1, 42) = 4.994, *p* = 0.032, *η*_*p*_^2^ = 0.105. and an interaction between congruency, alignment and group, *F*(1, 42) = 12.141, *p* = 0.001, *η*_*p*_^2^ = 0.224. However, there is no main effect of group, *F*(1, 42) = 1.065, *p* = 0.308, interaction between alignment and group, *F*(1, 42) = 0.012, *p* = 0.931, nor an interaction between congruency and group, *F*(1, 42) = 1.022, *p* = 0.318.

A post-doc 2 (congruency: congruent vs. incongruent) × 2 (alignment: aligned vs misaligned) repeated-measures ANOVA was conducted on Response Time for correct responses in participants with and without dyslexia separately. In participants with dyslexia, a significant main effect was found for congruency, *F*(1, 21) = 8.77, *p* = 0.009, *η*_*p*_^2^ = 0.295, as well as a significant interaction between congruency and alignment, *F*(1, 21) = 7.682, *p* = 0.012, *η*_*p*_^2^ = 0.278; however, only a marginal main effect of alignment was found, *F*(1, 21) = 3.784, *p* = 0.066. In typical readers, a significant main effect was found for both congruency, *F*(1, 21) = 6.431, *p* = 0.019, *η*_*p*_^2^ = 0.234, and alignment, *F*(1, 21) = 16.146, *p* = 0.001, *η*_*p*_^2^ = 0.435; however, no interaction between congruency and alignment was found, *F*(1, 21) = 2.430, *p* = 0.134. Post-doc *t*-tests showed that participants with dyslexia responded more slowly in the in the aligned than in the misaligned conditions in incongruent trials, *t*(21) = 2.607, *p* = 0.016, *d* = 0.56, whereas RTs in the aligned and misaligned conditions were similar in the congruent trials, *t*(21) = 0.1.048, *p* = 0.291. While dyslexic readers showed a faster response time in congruent than incongruent trials in aligned condition, *t*(21) = 3.534, *p* = 0.002, *d* = 0.75, they responded at a similarly speed in both congruent and incongruent trials in the misaligned condition, *t*(21) = 0.349, *p* = 0.731. In contrasts, the typical readers generally responded slower in aligned conditions than misaligned conditions in both congruent, t(21) = 3.294, p = 0.004, *d* = 0.68 and incongruent conditions t(21) = 4.588, p < 0.001, *d* = 0.98. While the typical readers showed a faster response time in congruent than incongruent trials in aligned condition, *t*(21) = 2.772, *p* = 0.011, *d* = 0.59, they responded at a similarly speed in both congruent and incongruent trials in the misaligned condition, *t*(21) = 0.877, *p* = 0.39.

**Figure 2:**
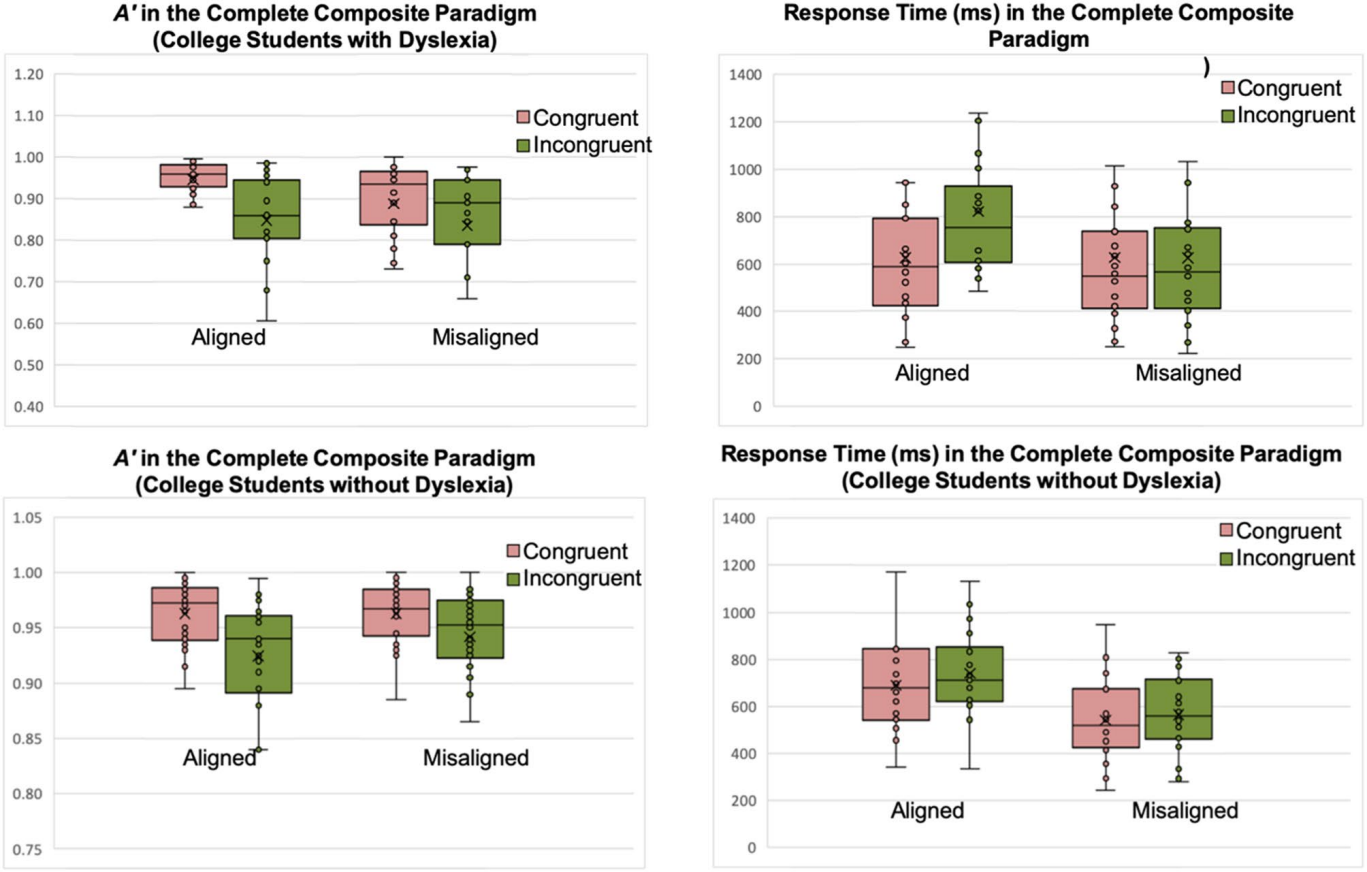
A′ and Response Time (shown in congruent and incongruent trials in both aligned and misaligned conditions) in dyslexics (top) and typically developing readers (bottom).

See Fig. 2 for an overall summary for the analysis of *A′* and RT.

**Figure 3:**
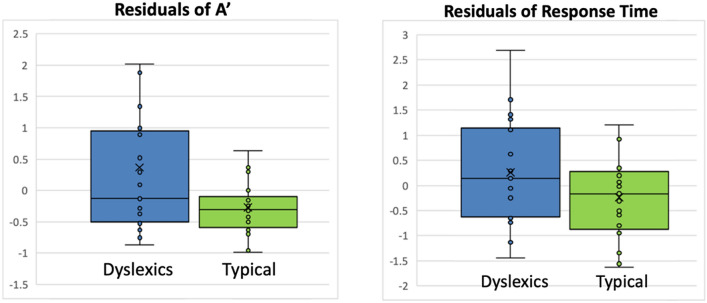
Centralized Residuals of *A′* (left) and response time (right) as computed with regression method^12^.

Using the traditional method of repeated-measures ANOVA that includes performance in misaligned trials in the analysis, only Response Time demonstrated that the participants with dyslexia perceived Chinese characters more holistically than the controls; while analysis of *A′* did not reveal differences in composite effect between participants with and without dyslexia. However, the inclusion of a misaligned condition is suggested to corrupt the validity of the measures in the congruent condition due to the baseline variance as a result of individual differences^12^. An additional analysis was conducted to examine the composite effect by subtracting *A′* score in the incongruent trials from congruent trials in both aligned and misaligned conditions, then regress the misaligned measure from the aligned measure to yield the residuals for each participant. This measure can take into account the performance in the misaligned conditions while correcting for individual baseline differences^12^. Independent sample t-test showed that dyslexic readers had a larger magnitude of *A′* residuals than the typical readers, t(42) = 2.226, p = 0.031, d = 0.69. With the same regression method, residuals of response time between aligned and misaligned conditions also differed between dyslexic and typical readers, t(42) = 2.020, p = 0.05, d = 0.64 (Fig. 3).

**Figure 4:**
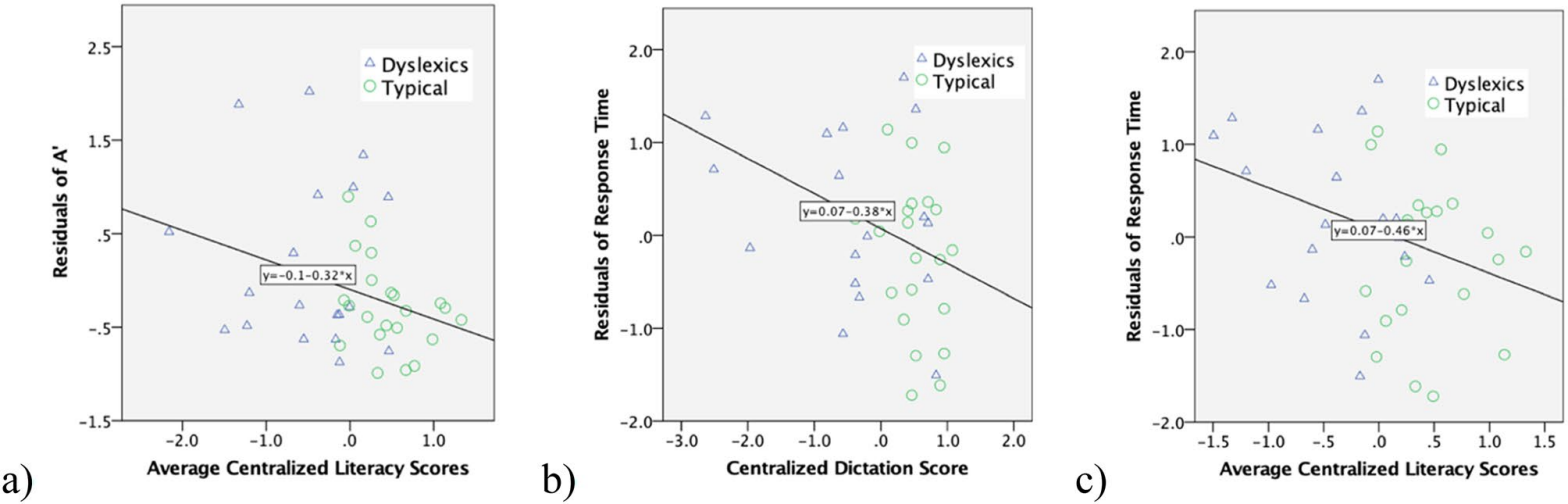
(**a**) Regression between residuals of *A′* and centralized literacy scores; (**b**,**c**) regression between residuals of response time and centralized literacy and dictation scores.

Table 2 presents the Pearson’s correlation analysis among the literacy measures and residual scores of *A′* and response time. A significant correlation was found between *A′* residual scores with overall Chinese literacy scores (see Fig. 4), but not between *A′* residual scores with specific Chinese literacy tasks. Response Time residuals correlate significantly with dictation scores and marginally significantly with overall Chinese literacy. These results suggest that the difference in holistic processing of Chinese characters (as measured by *A′* residual scores) between college students with and without dyslexia is associated with a general literacy performance in Chinese.

**Table 2:** Correlations Chinese literacy measures with residuals of A′.

	Overall Chinese literacy scores	Chinese word-reading	Chinese dictation	Chinese one-min word reading
Chinese word-reading	0.688**	–	–	–
Chinese dictation	0.654**	0.674**	–	–
Chinese one-min word reading	0.759**	0.188	0.038	–
A′ residual scores	− 0.319**	**− **0.240	**− **0.157	**− **0.269
Response times residual scores	− 0.302*	**− **0.259	− 0.329**	**− **0.061

*p = 0.058; **p < 0.05.

## Discussion

In this study a common face-perceptual expertise phenomenon—holistic processing—was used to compare how college students with dyslexia and those without processed Chinese characters. It is noted that a recent unpublished study found that English college students with dyslexia were more susceptible to congruency effect, though the effect was observed in both aligned and misaligned English word stimuli in the composite task^51^. However, the college participants with dyslexia in our present study showed a larger difference in congruency effect between aligned and misaligned condition when compared with typical readers, as shown in response time. Together with the analysis of *A′* using the regression method^12^, our results suggest that the dyslexic readers demonstrate stronger holistic processing effects in Chinese character recognition than the typically developing controls. Regression analyses suggest that this difference in holistic processing (as measured by residuals of *A′*) between participants with and without dyslexia is associated with a general Chinese literacy performance. It will be interesting for future studies to investigate the composite effect among dyslexics in Chinese and alphabetic languages to examine any differences and their underlying factors (e.g. visual properties or the type of learning experience). Nonetheless, here our results are consistent with prior studies which showed reduced holistic processing to be associated with expert Chinese character recognition^17,26^. The results from previous studies converged to show an analogous pattern of holistic processing between face and Chinese character recognition, in which drawing/writing experiences played a modulating role^26,27^. However, while reduced holistic processing has been identified as the core deficit in visual object/face recognition impairment, our results showed otherwise for Chinese character recognition^47,48^. Perhaps expertise in face recognition requires holistic processing—grouping all facial features together for efficient processing—as faces consist of homogenous configurations and features. However, true expertise in Chinese character recognition and writing requires the skills of isolating and identifying components, which result in reduced holistic processing for greater precision in character production^17,26,52^. It seems that typical literates who can read and write proficiently can readily ignore some configural information in Chinese characters, as such information is less important in character recognition (e.g., relative distances between character components^17,53^). Indeed, typical expert Chinese readers can switch between holistic and part-based processing depending on whether the recognition task relies on configural or featural information respectively^10,54,55^.

The unique role of character writing and copying skills in predicting Chinese reading development may account for the present findings. Holistic processing is a second-order configural processing in which both featural and spatial-distal information within an object are integrated and processed^7^. Hence, the stronger holistic processing effect of the dyslexic participants may suggest that they recognized characters with an over-dependence on the configural information of components. As the Chinese writing system is a logographic script which principally corresponds to meanings instead of phonology, learning to read and write Chinese as a logographic script was shown to hone visual-spatial skills in Chinese readers^39^. Indeed, early copying and writing abilities predict Chinese character recognition due to the deep-rooted practice of learning Chinese characters through copying^40^. Moreover, people with reading difficulties often demonstrate a marked discrepancy between word recognition and writing abilities due to Chinese character production being a more resource-intensive process than writing in alphabetic languages^46^. Impaired character-writing abilities in people with dyslexia may hinder the development of selective attention to individual character components (i.e. analytic processing)—this in turn hinders Chinese character recognition as it is an ability facilitated by sensitivity to the specific positions of components, radicals and structures within a character^49^. Indeed, it is also showed that Chinese reading involves both the decomposition and integration of the characters, suggesting that experts should be able to employ analytic or holistic processing flexibly, based on the task demand^49^. Consistently, holistic processing, as indicated by the residuals of response time, correlated negatively with writing performance in the current study. However, while it is suggested this reduced holistic processing effect can be explained by writing experiences/performances in expert Chinese readers, our result did not tease apart the effects of reading and writing abilities in dyslexic and normal participants as the dyslexic participants had a general lower performance in Chinese literacy^26^. Note that the study showed that Limited-writers were more holistic than novices, suggesting that holistic processing is still a marker of expertise in Chinese character recognition^26^. Given that both the students with dyslexia in this study and the Limited-writers studied had adequate experiences in character-recognition, perhaps the holistic processing in both Limited-writers and dyslexics also indicates experiences in reading^26^. Since writing practice is implemented regularly in the Chinese literacy curriculum in Hong Kong, students have learned to deemphasize unimportant configural information. However, students with dyslexia suffer from deficits that impair them from separating visually and attending selectively to character components. This in turn deters writing and hence hinders them from developing true expertise in character recognition as typically developing students can do. However, the focus of this study aims at looking at the group differences between readers with and without dyslexia, perhaps future studies can recruit a larger sample of typical readers. It is speculated that a larger sample of typical expert and/or intermediate readers who learned to read and write Chinese characters simultaneously will also reveal a significant negative correlation between holistic processing and Chinese word writing. Future studies may focus on the developmental trajectory of Chinese literacy development to confirm the above speculations.

Note that this study mainly used Chinese characters of top–bottom and left–right configurations, which contribute to 80 to 90% of the Chinese-character corpus^15^: top–bottom characters were chosen to simulate face recognition processes in which composite effects were observed in both faces and Chinese characters that are structurally top–bottom stimuli. However, there have also been speculations whether familiarity with the type of Chinese character structures would influence the composite effect, as left–right structure is the most dominant in the Chinese orthography, followed by the top–bottom structure^16^. Despite the above concerns, previous studies have shown similar patterns of composite effect across character types^62^. Hence, this study analyzed the composite effect by combining data from both top–bottom and left–right characters for a better generalization and representations of the Chinese character corpus. Nevertheless, preliminary data analysis of this study shows that only structurally left–right demonstrated a similar pattern in composite effect in response when both left–right and top–bottom characters were analyzed separately (see Supplementary Materials S1). Perhaps future studies can also address any possible differences in the processing of the two character structure types with a larger sample size with typical Chinese readers. Moreover, other characters of less frequent structure types, such as simple structure (single-radical) or enclosure, were not used in this study due to constraints of the composite paradigm design; these characters are not easily separable into 2 halves. Follow-up studies may address how characters of other structural types are processed in participants with dyslexia through a more carefully designed experiment.

In addition, in order to compare processes between face and word recognition, this study demonstrates holistic processing of single-characters as they are the smallest meaningful, pronounceable units of a Chinese word which can exist alone. Constituent components or radicals from Chinese characters are mostly unpronounceable; only through the combination of the components gives each Chinese character its identity. This property is similar to faces in which the combination of facial features gives each face its identity. In contrast, characters are free monosyllabic morphemes embedded in multicharacter Chinese words which can exist alone as meaningful and pronounceable units. Hence compound Chinese words are more easily and visually separable to readers—perhaps a reduced holistic processing is expected in both dyslexic and typical readers due to the above visual properties of multi-character Chinese words. Since deficits in orthographic sensitivity at the character-level is a common reoccurring and consistent marker in Chinese readers with reading difficulties^56^, this study investigates single character as the first step measure for assessing the role of holistic processing in Chinese character recognition. Though not the focus of the present research, future studies may investigate holistic processing of multicharacter words, as well as sentence.

Moreover, visual spatial skills deficits and abnormalities in visual selective attention have also been demonstrated in people with dyslexia in alphabetic languages^29,30^. This has been found to persist into adulthood^50^. Indeed, reading development in alphabetic languages moves from a global visual process to a more eventual local process of individual letters used by experts^57^. This phenomenon is associated with the need to attend selectively to individual letters in words for phonological decoding. Consistently, analysis of *A′* shows that the dyslexics in this study had a general congruency effect in both the aligned and misaligned conditions, suggesting a general abnormality of visual inhibition in word processing. However, note that the composite paradigm was adopted in this study in order to investigate the type of holistic processing here that is defined as the obligatory attention to all parts of a stimuli as a whole. In this study, a composite effect was revealed by response time and regression scores of *A′.* This suggests that the visual processing abnormality in Chinese character recognition observed in the dyslexics can be better explain by holistic processing effect than response inhibition. Nevertheless, it will be interesting for future studies to investigate Chinese character recognition processes using different paradigms that are associated with perceptual expertise effects. Future studies can also investigate the possible role of holistic processing in English word recognition with the complete composite paradigm.

To conclude, this study has shown that adults with dyslexia processed Chinese characters more holistically than did the typical controls. This suggests that the adult dyslexics had persistent deficits in selectively attending to character components when processing Chinese characters, hindering the development of expert reading and writing skills. This study has also provided evidence that college students with dyslexia in Chinese still encountered difficulties in reading and writing due to persistent deficits in their literacy-related cognitive abilities. These adults still required further support in their college learning, and more research should be conducted to order to understand their cognitive profiles further. However, because the number of students with dyslexia enrolled in universities in Hong Kong is extremely low, together with the low diagnostic rate of dyslexia in the recent past in Hong Kong, it has been difficult to study dyslexia in adult Chinese samples^58,59^. Hence, we are the first to report the perceptual differences between typically developing and dyslexic participants in college by investigating holistic processing of Chinese character recognition.

## Methods

### Participants

Twenty-two participants with dyslexia and twenty-two typically developing students (15 males in each group) were recruited from universities in Hong Kong. All participants with dyslexia had been diagnosed since high school or prior. All of them were right-handed according to the Edinburgh Handedness Inventory and had normal or corrected-to-normal vision. The two groups were matched by age, *t*(42) = 1.352, *p* = 0.184 (Controls: *M* = 21.92, *SE* = 0.63; Dyslexics: *M* = 20.85, *SE* = 0.49). All procedures were carried out in accordance with relevant guidelines and regulations from Human Research Ethics Committee, The Education University of Hong Kong. All experimental protocols were also approved by Human Research Ethics Committee, The Education University of Hong Kong. Informed consent was obtained from all participants (and from a parent and/or legal guardian for participants of age under 18). Informed consent for open-access publication of all identifying images in this paper has been obtained.

### Holistic processing

With reference to the previous experiment, a complete composite paradigm was designed to measure holistic processing with 160 pairs of real Chinese characters with a top–bottom and left–right configuration, in Ming font^17^.

**Figure 5:**
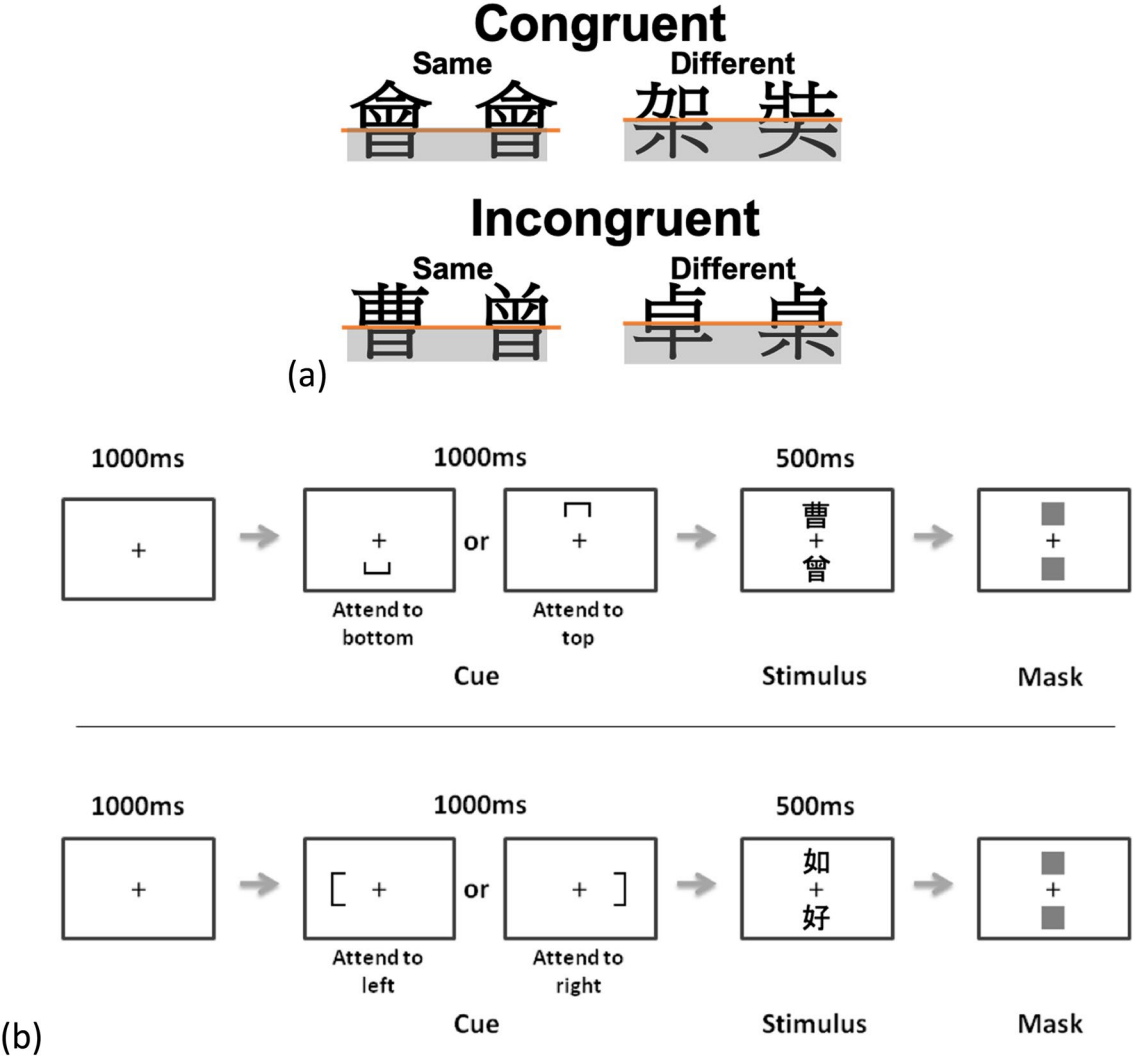
Illustration of stimulus pairs in the complete composite paradigm (**a**) and trial sequences (**b**). In (**a**), the four conditions used in the paradigm are shown; the attended components are shaded in grey. In (**b**), a 1000 ms central fixation cross precedes each trial, followed by a cue either below or above the cross, or to the left or right of it, to indicate which halves (top or bottom/left or right) of the characters the participant should attend to in the subsequent display.

A pair of Chinese characters were displayed to the participants in each trial, instructing them to attend only to either their bottom or top halves and judge whether they were the same. In congruent trials, the two characters in a pair were either totally the same (Congruent-Same) or different (Congruent-Different) in both attended and irrelevant halves; in incongruent trials, either the top halves of both the characters were different while the bottom halves were the same (Incongruent-Same), or the top halves were the same while the bottom halves were different (Incongruent-Different). As illustrated in Fig. 5a, the characters were presented in all four conditions with equal stroke numbers and ranged in a frequency from medium to high^60^. The complete (instead of partial) composite paradigm was used here in order to avoid response biases when the irrelevant halves were always to be different^9^.

Every trial began with a center-fixation cross for 1000 ms, followed by a symbol indicating which halves of the Chinese characters the participants should pay attention to. Then, two Chinese characters appeared, one below and one above the fixation cross, for 500 ms. After looking at both Chinese characters, the participants needed to judge as accurately and quickly as possible whether the indicated halves of the characters were the same (Fig. 5b). The participants responded by pressing the appropriate buttons on the keyboard. Their discrimination sensitivity *A′* was measured as follows:

$$A^{\prime} = 0.5 + \left[ {sign(H - F)\frac{{(H - F)^{2} + \left| {H - F} \right|}}{4\max (H,F) - 4HF}} \right]$$

where “H” indicates the hit rate and “F” is the false alarm rate. A′ is a nonparametric measure of sensitivity which is bias-free; d′ was not used because when homogeneity of variance and normality are not assumed, the response biases may affect its measurement^17,26^. Their response time for correct responses in each trial was also recorded. Holistic processing was measured by the A′ or response time difference between congruent and incongruent trials—stronger holistic processing was indicated by a more positive value. Note that the sequential matching paradigm is another common procedure for the composite task which requires the first stimuli to be retained in the working memory before the second stimuli appears for the participants to make a judgement. We adopted simultaneous paradigm in order to reduce participants’ the memory load^17,26,61,62^. Nevertheless, either sequential or simultaneous matching paradigms should yield similar effects^25,63^.

**Figure 6:**
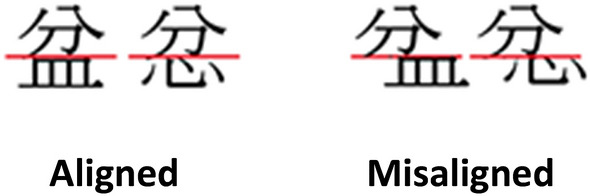
Example of the stimulus pairs in aligned and misaligned conditions.

Unlike in the partial composite paradigm in which a misaligned condition (i.e. the top halves misaligned from the bottoms halves of the stimuli) must be administered^64^, this study adopted the complete composite paradigm in which holistic processing is indicated by the difference in performance between congruent and incongruent trials without the need of misaligned trials (see Fig. 6)^17,26,65^. Indeed, one advantage of including a misaligned condition is to rule out the possibility of congruency effect due to response inhibition. However, our participants were all adults studying in college, and the performance difference between groups in incongruent trials was unlikely to be due to differences in inhibition control^26^. It is also demonstrated that the congruency effect in Chinese character processing observed in novice adults disappeared in misaligned trials, suggesting that the effect was indeed associated with the inability to attend selectively to aligned character parts instead of response inhibition difficulties^17^. Nevertheless, we have included a condition in which misalignment indeed diminished the congruency effect in both groups (see “Results”). Aligned and Misaligned conditions were presented block-wise in a randomized sequence.

### Non-verbal intelligence

All participants in this study were enrolled in bachelor’s programs in Hong Kong government-funded universities, and hence dyslexic and typical participants should have comparable intelligence levels. To control for the effect of intelligence on holistic processing and literacy performance, nonverbal intelligence was assessed using the 9-item subset of Raven’s standard progressing matrices (9-item Raven’s)^66,67^. It is noted that the 9-item Raven’s was validated only for research purposes and not for intelligence screening^67^, hence was adopted to avoid excessive clinical testing on our participants.

### Chinese literacy performance

To date, there is no standardized diagnostic tool for dyslexia in Chinese for Hong Kong adults over 16 years of age, therefore the stimuli to assess Chinese literacy and literacy-related cognitive performances were adopted from the Hong Kong Test of Specific Learning Difficulties in Reading and Writing for Primary School Students—Third Edition [HKT-P(III)]^56^. Since this study had no diagnostic purpose, the results from the tests only gave us a comparison between the literacy performances of the college participants with dyslexia and those without. Since the symptoms of dyslexia in Chinese may be manifested in weakness in either reading or writing, or both, the scores from the three literacy subtests from HKT-P(III)—Word reading task, One-minute word reading task and Dictation task—were clustered and analyzed as a single composite score in the literacy domain by averaging the z-scores of the three literacy subtests^56^ (i.e. an *averaged centralized literacy score*):

#### Chinese word reading

This was a subtest from HKT-P(III) that assessed word reading accuracy. The participants were instructed to read aloud a list of 120 two-character Chinese words presented in ascending order of difficulty. One point was given for each word read correctly.

#### One-minute word reading

This was a timed subtest from HKT-P(III) that measured fluency in Chinese word reading. 120 simple high-frequency Chinese words were displayed in 20 rows of 6 words each. The participants were instructed to read aloud as many of the words as possible in one minute. One point was given for each word read correctly.

#### Chinese word dictation

This subtest from HKT-P(III) assessed accuracy in word recall and writing in Chinese. The participants were instructed to write down 42 two-character words after hearing a word read to them in each trial by the experimenter. One point was given for each correct character written by the participants.

#### Rapid digit naming

This subtest from HKT-P(III) measured rapid automatized naming of highly familiar visual stimuli, a task that closely predicts literacy performance across difference languages^68^. The participants were presented with a matrix of 8 rows of 5 digits arranged in random sequences, and were instructed to name aloud the digits in serial order as quickly and as accurately as possible. They were instructed to perform the task twice, and the score was the average time taken for the two trials.

## Supplementary Information

Supplementary Information
