# Supplementary material for: Holistic processing of Chinese characters in college students with dyslexia
Source: Sci Rep. 2021 Jan 21;11:1973. doi: 10.1038/s41598-021-81553-5 (PMC7820259; doi:10.1038/s41598-021-81553-5)
Supplement: Supplementary file 1 — Supplementary Information [file 41598_2021_81553_MOESM1_ESM.docx]

**Holistic Processing of Chinese Characters in College Students with Dyslexia**

**Ricky Van-yip Tso^a,b,*^, Ronald Tsz-chung Chan^a^, Yin-fei Chan^a^, and Dan Lin^a^**

^a^Department of Psychology, The Education University of Hong Kong, Hong Kong

^b^Psychological Assessment and Clinical Research Unit, The Education University of Hong Kong, Hong Kong

*[rvytso@eduhk.hk](mailto:rvytso@eduhk.hk)

**Supplementary Material**

*Summary of 2x2 (congruency x alignment) repeated-measures ANOVA*

*of performance for each character structure*

| **Left-right structures** | *F* | | *p* | | *η_p_²* | |  | |  |
| --- | --- | --- | --- | --- | --- | --- | --- | --- | --- |
| ***Dyslexics*** |  | |  | |  | |  | |  |
| RT |  | |  | |  | |  | |  |
| Congruency | 15.306* | | .001 | | .422 | |  | |  |
| Alignment | 2.664 | | .118 | | .113 | |  | |  |
| Congruency x Alignment | 12.498* | | .002 | | .373 | |  | |  |
| A’ |  | |  | |  | |  | |  |
| Congruency | 7.164* | | .014 | | .254 | |  | |  |
| Alignment | .811 | | .378 | | .037 | |  | |  |
| Congruency x Alignment | .617 | | .441 | | .029 | |  | |  |
| ***Typical Readers*** |  | |  | |  | |  | |  |
| RT |  | |  | |  | |  | |  |
| Congruency | 4.494* | | .046 | | .176 | |  | |  |
| Alignment | 4.892* | | .038 | | .189 | |  | |  |
| Congruency x Alignment | 1.280 | | .271 | | .057 | |  | |  |
| A’ |  | |  | |  | |  | |  |
| Congruency | 4.008 | | .058 | | .160 | |  | |  |
| Alignment | .899 | | .354 | | .041 | |  | |  |
| Congruency x Alignment | .036 | | .851 | | .002 | |  | |  |
| **Top-bottom structures** | | *F* | | *p* | | *η_p_²* | |  | |
| ***Dyslexics*** | |  | |  | |  | |  | |
| RT | |  | |  | |  | |  | |
| Congruency | | 1.703 | | .206 | | .075 | |  | |
| Alignment | | 2.706 | | .115 | | .114 | |  | |
| Congruency x Alignment | | .162 | | .692 | | .008 | |  | |
| A’ | |  | |  | |  | |  | |
| Congruency | | 16.006* | | .001 | | .433 | |  | |
| Alignment | | 2.059 | | .166 | | .089 | |  | |
| Congruency x Alignment | | 2.238 | | .150 | | .096 | |  | |
| ***Typical Readers*** | |  | |  | |  | |  | |
| RT | |  | |  | |  | |  | |
| Congruency | | 2.395 | | .137 | | .102 | |  | |
| Alignment | | 14.094* | | .001 | | .402 | |  | |
| Congruency x Alignment | | 1.046 | | .318 | | .047 | |  | |
| A’ | |  | |  | |  | |  | |
| Congruency | | 24.263* | | <.001 | | .536 | |  | |
| Alignment | | .289 | | .597 | | .014 | |  | |
| Congruency x Alignment | | 2.842 | | .107 | | .119 | |  | |
